# To the Dental Profession

**Published:** 1857

**Authors:** 


### TO THE DENTAL PROFESSION.

GENTLEMEN:—A year has rolled round since I made my debut (on my own responsibility) in the Dental Furnishing Business. I am still "alive and kicking," increasing my facilities as rapidly as possible to manufacture and furnish supplies to meet the growing demand, the *prediction* by some of my "friends" that I "could not stand a year," to the contrary notwithstanding.

I have now been eight years engaged in the *Dental Depot Business*—seven years in the employment of others, and one year for myself; how well I have performed my duties, I will leave for others to say. I know that I have my faults and weaknesses, like others; but my constant effort has been to do as near right as I could, and if by accident or otherwise, I fell short of my *whole duty*, have ever been ready to make just and satisfactory amends.

Like all others who commence a business of *minutia*, I have had many difficulties to contend with and obstacles to surmount. Want of capital, having to employ *raw* hands, besides a rather *peculiar* competition, has made it necessary to attend to almost every transaction in person, for which an eight years' experience in the details of the business has qualified me well, and gives me unequaled advantages for comprehending the wants and supplying the precise articles desired.

Probably the best evidence on this score is the fact that I have on my list of regular customers nearly all of the first class Dentists in the West and South—men who know what they want, and expect proper business attention, and can *appreciate* the efforts made in their behalf.

As I stated in my first circular, I have engaged in this business for the purpose of *making money*, *not* by selling goods at high prices, and making large profits, but by furnishing the profession with goods of *superior quality* at the *lowest prices*.

By keeping a large and carefully selected stock, and procuring every new and useful invention or improvement as early as practicable, thus giving the advantages of the Eastern markets, added to the products of the labor, skill, and ingenuity of the West, *all* in *one establishment*, easy and convenient of access; thus making it the interest of *all* to send their orders here in preference to any other place.

If these efforts on my part are properly appreciated, and I believe they will be, many of those who formerly sent their orders

East will hereafter send here, and thus increase the amount of business to be done; and just in proportion to the support and encouragement that is shown, in the same ratio do you enable me to sell cheap, and furnish you with goods.

I can do a business of $100,000 a year at about the same expense as $20,000. The rent, light, fuel, etc., are the same, and, of course, if I sell largely, I can afford to sell at less profits than I could with a small business. My business has expanded far beyond my anticipations, and is growing rapidly. I would here return my thanks for the kindness, appreciation, and liberality of the patronage heretofore received, and hope to MERIT its continuance, by every effort on my part to fulfill the wishes and expectations of the profession.

Where the field is sufficiently large—as it is here—an honorable competition is beneficial to all parties concerned, the profession desire it, and are especially interested; but they should be very CAREFUL, whilst they are endeavoring to BUY CHEAP, that they do not encourage a species of competition which is an injury to all. As no man can do business for nothing, or sell goods for less than cost, where such things are done, there must be deception in the article itself, or the amount must be made up on something else; otherwise such competition can only be *temporary*, as business men will not long pursue a losing business. It would be well, therefore, to be careful not to sacrifice a permanent advantage to a temporary one. If goods are sold in Cincinnati as cheap as in New York and Philadelphia—where there has been competition for years—the Dentist should be satisfied, as it could hardly be expected that they could be afforded lower.

